# An *In-situ* Real-Time Optical Fiber Sensor Based on Surface Plasmon Resonance for Monitoring the Growth of TiO_2_ Thin Films

**DOI:** 10.3390/s130709513

**Published:** 2013-07-23

**Authors:** Yu-Chia Tsao, Woo-Hu Tsai, Wen-Ching Shih, Mu-Shiang Wu

**Affiliations:** Graduate Program in Electro-Optical Engineering, Tatung University, 40 Chongshan N. Rd., 3rd Sec. Taipei 104, Taiwan; E-Mails: whtsai@ttu.edu.tw (W.-H.T.); wcshih@ttu.edu.tw (W.-C.S.); mswu@ttu.edu.tw (M.-S.W.)

**Keywords:** surface plasmon resonance, optical fiber sensor

## Abstract

An optical fiber sensor based on surface plasmon resonance (SPR) is proposed for monitoring the thickness of deposited nano-thin films. A side-polished multimode SPR optical fiber sensor with an 850 nm-LD is used as the transducing element for real-time monitoring of the deposited TiO_2_ thin films. The SPR optical fiber sensor was installed in the TiO_2_ sputtering system in order to measure the thickness of the deposited sample during TiO_2_ deposition. The SPR response declined in real-time in relation to the growth of the thickness of the TiO_2_ thin film. Our results show the same trend of the SPR response in real-time and in spectra taken before and after deposition. The SPR transmitted intensity changes by approximately 18.76% corresponding to 50 nm of deposited TiO_2_ thin film. We have shown that optical fiber sensors utilizing SPR have the potential for real-time monitoring of the SPR technology of nanometer film thickness. The compact size of the SPR fiber sensor enables it to be positioned inside the deposition chamber, and it could thus measure the film thickness directly in real-time. This technology also has potential application for monitoring the deposition of other materials. Moreover, *in-situ* real-time SPR optical fiber sensor technology is in inexpensive, disposable technique that has anti-interference properties, and the potential to enable on-line monitoring and monitoring of organic coatings.

## 1. Introduction

Over the past decade, surface plasmon resonance (SPR) has been used in a wide range of chemical and biological sensing applications. SPR is a charge density oscillation that may exist at the interface of two media with dielectric constants of opposite sign, for instance, a metal and a dielectric. The excitation of a surface plasmon wave leads to the appearance of a dip in the measured intensity of reflected light, which must be considered in determining the sensitivity of SPR sensing. The first use of SPR in prism coupling was proposed by Kretschmann in 1968 [1]. In the early years after the basic theory of the SPR phenomenon was presented by Kretschmann, some attempts were made to develop an SPR sensor based on the optical interaction effect. Since then, surface plasmons have been studied extensively, and their major properties have been assessed [2]. The sensitivity of an SPR sensor is defined as the derivative of the monitored SPR parameter, which can detect changes of the refractive index. Several detection schemes have been demonstrated for SPR prism-based sensors; they include angular interrogation [3], wavelength interrogation [4], and intensity measurement [5].

Since Jorgenson and Yee proposed using optical fibers for SPR sensing in 1993 [6], many types of optical fiber sensor have been proposed, including single-mode dip fibers [7], single-mode D-type fibers [8], and D-shaped fibers [9]. In recent years, studies of SPR sensing systems have been focused on the attenuated total reflection geometry obtained by use of prism-coupling optics. However, those systems of optical fiber sensors need bulky structures as well as complicated signal processing to improve their high sensitivity. In contrast, the side-polished multimode fiber sensor provides a simple structure and system for chemical and biological sensing with high sensitivity in wavelength interrogation [10,11], and also has a high detection limit for biomolecules [11,12]. Time-dependent measurements of SPR fiber sensors are important for applications in biosensing and in environmental monitoring, and the sensitivity of intensity measurements with side-polished fiber sensors has been demonstrated [13,14].

Most of today's available techniques are restricted to certain type of films and many have difficulties with performing measurement *in-situ*. Various thin film measurement methods, such as surface profilometry and resistivity measurement are very difficult to carry out *in-situ*, and analysis of film growth rate is usually performed after the deposition run. Currently, ellipsometry is preferred as an *in-situ* monitoring tool [15–17]. However, this method lacks the ability to measure opaque films. Quartz crystal monitors are also available as an *in-situ* monitoring tool, but they only offer an indirect measurement of the thickness of a film grown on a surface [18,19]. To overcome these limitations, we have proposed a new *in-situ* monitoring technique utilizing a side-polished multimode optical fiber sensor based on surface plasmon resonance to measure the thickness of deposited nano-thin films.

## 2. Experiment

### 2.1. The Side-Polished SPR Optical Fiber Sensor

**Figure 1.**
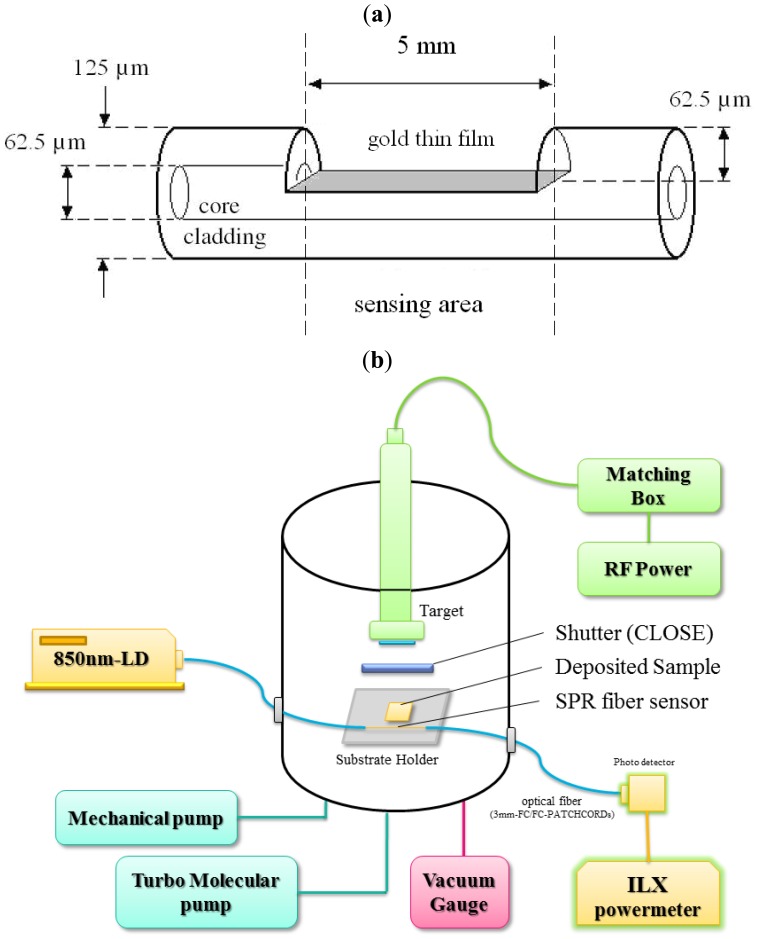
(**a**) Schematic diagram of the SPR optical fiber sensor with sided-polished structure. (**b**) Schematic diagram of the PVD sputtering system and SPR optical fiber sensing system.

The SPR optical fiber sensor consists of a gold thin film and a side-polished structure. This sensor outputs the SPR response spectrum and provides a high detection limit for biomolecules [11–13]. The optical fiber sensor based on surface plasmon resonance with side-polished structure is shown in Figure 1a. The graded-index multi-mode fiber with 62.5 μm core diameter and 62.5 μm cladding thickness, fabricated by Prime Optical Fiber Corporation (POFC), was side-polished to make an SPR optical fiber sensor. For high yield rate polishing processes, a silicon V-groove must be fabricated to hold the bare fibers. Thus, we grew the oxides layer on a 4-inch silicon (1 0 0) wafer and used photolithography to etch a SiO_2_ channel with 25% HF. The V-groove channel was etched by 45.3% KOH; the channel length and width were 5 mm and 125 μm, respectively. The multimode fiber was mounted on the V-groove holder with photoresist and monitored by optical microscopy. After the photoresist became hard, we polished the fibers that were embedded in the wafer using polishing diamond films with grain sizes of 6, 1 and 0.1 μm in turn. To increase the sensitivity of the SPR measurements, the length of the polished surface who set to 5 mm and the depth to 62.5 μm for the fundamental mode region. The dimensions of the polished surface, length and breadth, were 5 mm and 62.5 μm, and the depth of 62.5 μm was confirmed by optical microscopy (Olympus, BH, Tokyo, Japan).

The gold thin film was deposited on the polished surface by a DC sputtering system (ULVAC Co., Kanagawa, Japan). During the deposition process, the vacuum chamber was evacuated to 4 × 10^−2^ torr, and the background pressure was 2 × 10^−5^ torr. The gold thin film was approximately 40 nm thick, and was observed using an SEM (scanning electron microscope, JEOL 7000, JEOL, Tokyo, Japan). All of these optical parts were manufactured by New Product Div., Forward Electronics Co., New Taipei City, Taiwan.

### 2.2. TiO_2_ Sputtering System and SPR Optical Fiber Sensing System

As shown in Figure 1b, the *in-situ* TiO_2_ sensing system of the SPR optical fiber sensor consists of a light source (850 nm-LD), a photodetector, an ILX power meter (with personal computer), an SPR fiber sensor, and an RF planar magnetron sputtering system (ANELVA SPF-210HS, Kanagawa, Japan). This system was used to demonstrate in-situ real-time monitoring of the growth of TiO_2_ thin film based on the variation of the transmitted intensity of the SPR optical fiber sensor. The components are connected with 3-mm FC/FC-PATCHCORDs, which have a small insertion loss (<0.05 dB) and back reflection (<−55 dB) and are manufactured by General Optics Corporation (Zhongli, Taiwan). Because the surface plasmon can be excited by 850 nm light [12] via a suitable optical fiber, a light source (850 nm VCSEL MM Module, Appointech. Inc., Hsinch, Taiwan) with temperature-control is used. The light source also has high coupled power and reliability with pigtail-connected fiber. The Si-photodetector (Electro-Optics Technology, Inc., Traverse City, MI, USA) can monitor the output of an externally modulated cw laser. An ILX powermeter (OMM-6810B, ILX Lightwave, Irvine, CA, USA) suitable for a wavelength range of 350–1,700 nm, was also used. The sensitivity of the optical power meter is approximately 0.01 pW, and a personal computer was used to acquire data from measurements of the transmitted power.

An RF planar magnetron sputtering system (ANELVA SPF-210HS) was used to prepare the TiO_2_ thin film for depositing. A 99.9% pure TiO_2_ target disc, 2 inches in diameter, was used as the sputtering target. The chamber was pumped down to 1 × 10^−6^ torr using a turbo molecular pump before the sputtering gas (Ar) was introduced into the chamber through the mass flow controllers and controlled by the main valve of the pumping system. In all experiments, the target was pre-sputtered for 30 min under 60 W RF power before the actual sputtering to remove any contamination on the target surface. We fixed the target-to-substrate spacing at 50 mm, the deposition time to 30 min, and deposition pressure to 4 × 10^−4^ torr for the in-situ real time SPR monitoring of the growth of TiO_2_ thin films.

### 2.3. Configuration of the SPR Optical Fiber Sensor and Deposited Sample

**Figure 2.**
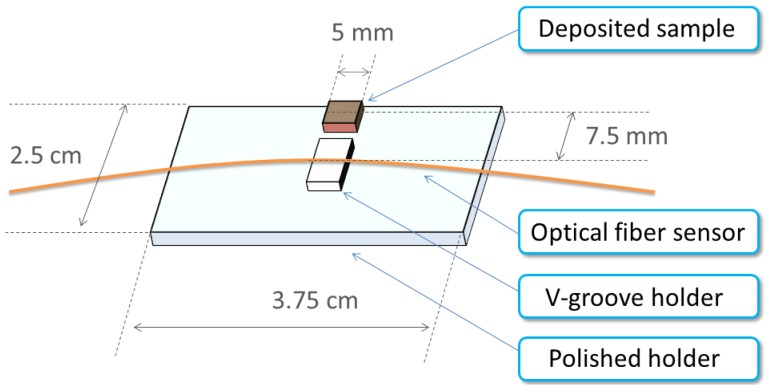
Schematic diagram of the setup for the SPR optical fiber sensor and deposited sample.

**Figure 3.**
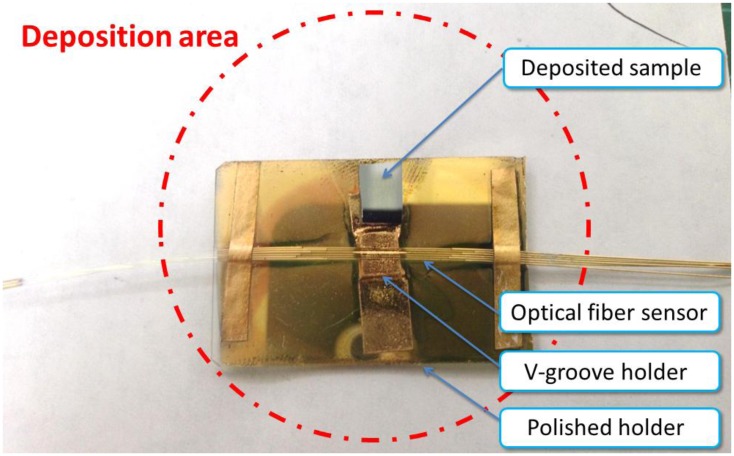
Deposition area and locations of the SPR optical fiber sensor and deposited sample.

The detection area, side-polished surface, of the optical fiber sensor and the sputtering direction must be in the same direction, particularly in the horizontal plane of the deposited sample. The manufacture of the SPR optical fiber sensor is described in Section 2.2. The multimode optical fiber is installed in a V-groove on a polished holder to avoid the breaking the optical fiber between the edges of V-groove during the polishing step. To determine the direction with the SPR optical fiber sensor, the deposited sample is set on the polished holder; as shown in Figure 2. The distance between the centers of the deposited sample and optical fiber sensor is 7.5 mm. Figure 3 shows that the polished holder (2.5 **x** 3.75 cm) contains the optical fiber sensing area and deposited sample all in the 2-inch radius TiO_2_ deposition area.

## 3. Results and Discussion

**Figure 4.**
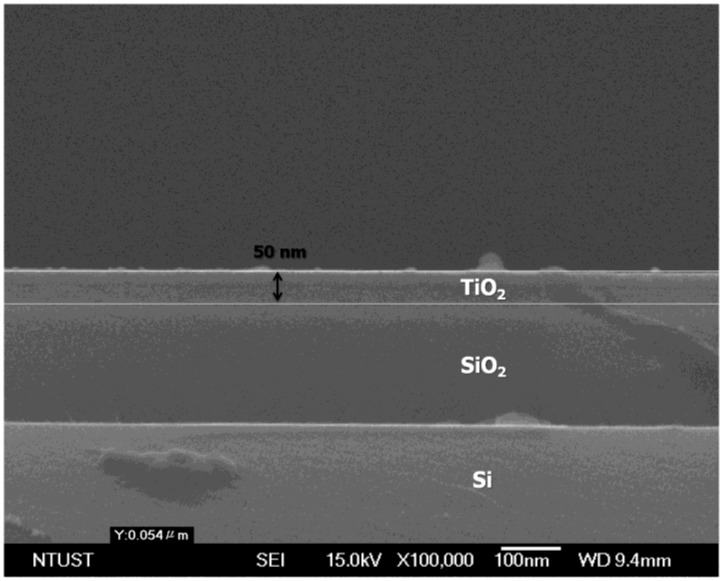
The thickness of the 50 nm-TiO_2_ thin film with 30 min. deposition time as observed by FE-SEM.

In this study, we compared of the deposited sample with the SPR response of an optical fiber to demonstrate the possibility of in-situ real-time monitoring. To monitor the growth of TiO_2_ directly, the SPR optical fiber sensor was positioned near the deposited sample in the TiO_2_ sputtering system, as shown in Figures 2 and 3. The thickness of TiO_2_ thin film deposited in 30 min, measured using an FE-SEM (High Resolution Field-Emission Scanning Electron Microscope, JEOL JSM-6500F) is 50 nm, as shown on Figure 4.

**Figure 5.**
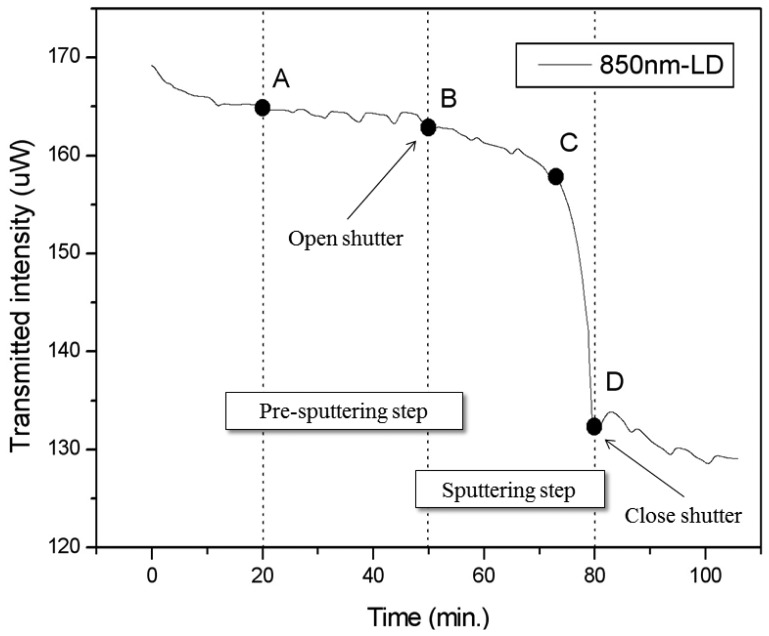
Dynamic experimental results for an SPR optical fiber sensor with 850 nm-LD monitoring the growth of deposited TiO_2_ thin film in-situ in real time. The pre-sputtering process occurs between points A and B. The sputtering process occurs between points B and D.

The SPR response while monitoring the growth of TiO_2_ thin film in real time is shown in Figure 5. The X-axis is time in minutes, and the Y-axis is the transmitted intensity. The transmitted intensity is the output power at the end of the optical fiber as measured by the ILX powermeter. There are two major steps in the sputtering process: pre-sputtering and sputtering. We use pre-sputtering to remove contaminant particles from the target surface; the transmitted intensity was not affected in this process. The location of shutter is between target and substrate. When the shutter is close, there will be no anything deposited on the substrate. After pre-sputtering the shutter is opened for the deposition of TiO_2_ thin film on the substrate. As shown in Figure 5, the sputtering time is 30 min.

The SPR response measured in real-time during the 30 min pre-sputtering process (between points A and B in Figure 5) is approximately 163.85 μW (164.5 μW ∼ 163.2 μW). The influence of the SPR optical fiber sensor cause less than 1.3 μW variation in the vacuum chamber, and does not significantly affect the signal of SPR optical fiber sensor in the vacuum chamber. After opening the shutter for TiO_2_ sputtering, the SPR response monitored in real-time for 30-min of sputtering changes from 162.87 μW at point B to 132.33 μW at point D in Figure 5.

The SPR response shows that TiO_2_ was deposited on the surface of the optical fiber. It also shows two distinct steps. It is well-known that the increased thickness of thin films is the factor that causes the SPR dip to shift to a longer-wavelength [11]. The most sensitive part of the SPR response occurs at the center of the SPR dip in wavelength. The first step of the SPR response, from point B to point C, shows minor variation and changes due to noise from 162.87 μW to 158.02 μW. The second step of the SPR response, from point C to point D, displays large changes from 158.02 μW to 132.33 μW. The TiO_2_ sputtering finished after the shutter was closed at point D. This result shows that the deposition of TiO_2_ thin films can be monitored *in-situ* in real-time by the SPR response of an optical fiber sensor.

**Figure 6.**
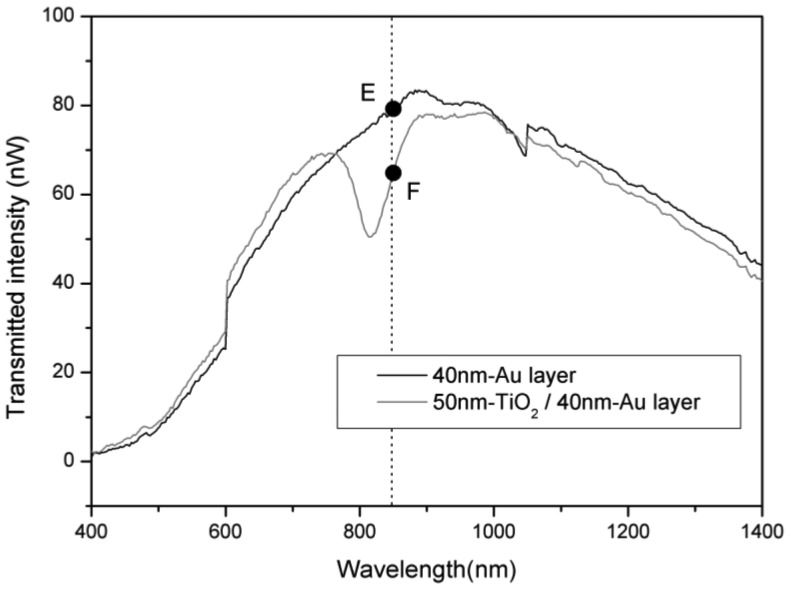
The spectroscopic SPR response with a halogen light source for a 50 nm layer of TiO_2_ deposited on an Au layer. The black and gray lines are the SPR spectra for a 40 nm-Au layer and a 50 nm-TiO_2_/40 nm-Au layer, respectively, as measured by OSA. The dashed line is set to calculate the change of the intensity at 850 nm.

The spectroscopic SPR response in spectra, measured by halogen light and an optical spectrum analyzer (OSA), is well-known [10,11,20]. The SPR spectra for layers of Au and TiO_2_/Au are shown on Figure 6. The black and gray lines are the SPR spectra for a 40 nm-Au layer and a 50 nm-TiO_2_/40 nm-Au layer, respectively, which were measured by optical spectrum analyzer (OSA, AQ6315A, Ando Electric Co., Ltd. Tokyo, Japan). The SPR spectral responses show that the transmitted intensity at 850 nm from E to F drops from 78.97 nW down to 63.77 nW. In other words, the transmitted intensity at 850 nm after 50 nm TiO_2_ sputtering has dropped to 80.75% of the value before sputtering.

The real-time measurement of the SPR response shows a drop in transmitted intensity during sputtering to 81.24% of the pre-sputtering value, as seen in Figure 5 between points B and D. Thus, the same trend is seen in the real-time *in-situ* measurements as in the spectroscopic measurements. The SPR transmitted intensity response changes in real-time approximately 18.76% for a deposition of 50 nm thickness of TiO_2_ thin film. We expect that thicknesses of coated TiO_2_ thin film will correspond to a different SPR dip wavelengths. These measurements display the potential for real-time monitoring of nanometer film thickness with SPR optical fiber sensor technology. Further experiments and measurements investigating different coating film thicknesses and their corresponding dip wavelengths are under way.

## 4. Conclusions

An *in-situ* thin film monitoring technique based on surface plasmon resonance and utilizing a side-polished multimode optical fiber sensor is proposed to measure the thickness of deposited nano-thin films. In this study, we positioned the SPR optical fiber sensor near the deposited sample in the TiO_2_ sputtering system for real-time monitoring. The 850 nm-LD light source is suitable for exciting surface plasmon resonance in an optical fiber sensor. The sputtering process deposited TiO_2_ on the material surface to change the refractive index, the effect of which was measured in real time by the SPR optical fiber sensor by monitoring the transmitted intensity of the SPR response. The decrease of the SPR response seen in real-time was related to the thickness of the TiO_2_ thin film deposited and showed the same trend as the spectral SPR response. The SPR transmitted intensity measured in real-time changes by approximately 18.76% for a deposition of a 50 nm of TiO_2_ thin film. We have shown the potential for real-time monitoring nanometer film thickness with the SPR response of optical fiber sensors. The compact size of the SPR fiber sensor allowed it to be set into the deposition chamber and thus measure directly in real-time. This technology also has the potential for application in other material deposition monitoring. Moreover, *in-situ* real-time SPR optical fiber sensor technology is inexpensive and disposable, has anti-interference properties, and has the potential to be used as an on-line monitor or organic coating monitor.
